# Copy Number Variations Due to Large Genomic Deletion in X-Linked Chronic Granulomatous Disease

**DOI:** 10.1371/journal.pone.0027782

**Published:** 2012-02-27

**Authors:** Takashi Arai, Tsutomu Oh-ishi, Hideaki Yamamoto, Hiroyuki Nunoi, Junji Kamizono, Masahiko Uehara, Takeo Kubota, Takuya Sakurai, Takako Kizaki, Hideki Ohno

**Affiliations:** 1 Department of Clinical Research, Saitama Children's Medical Center, Saitama, Japan; 2 Division of Infectious Disease, Saitama Children's Medical Center, Saitama, Japan; 3 Department of Pediatrics, Faculty of Medicine, University of Miyazaki, Miyazaki, Japan; 4 Department of Pediatrics, Kitakyusyu City Yahata Hospital, Kitakyushu, Japan; 5 Department of Pediatrics, Health Insurance Hitoyoshi General Hospital, Hitoyoshi, Japan; 6 Department of Epigenetic Medicine, Interdisciplinary Graduate School of Medicine and Engineering, University of Yamanashi, Chuo, Japan; 7 Department of Molecular Predictive Medicine and Sport Science, Kyorin University, School of Medicine, Mitaka, Japan; South Texas Veterans Health Care System and University Health Science Center San Antonio, United States of America

## Abstract

Mutations in genes for any of the six subunits of NADPH oxidase cause chronic granulomatous disease (CGD), but almost 2/3 of CGD cases are caused by mutations in the X-linked *CYBB* gene, also known as NAD (P) H oxidase 2. Approximately 260 patients with CGD have been reported in Japan, of whom 92 were shown to have mutations of the *CYBB* gene and 16 to have chromosomal deletions. However, there has been very little detailed analysis of the range of the deletion or close understanding of the disease based on this. We therefore analyzed genomic rearrangements in X-linked CGD using array comparative genomic hybridization analysis, revealing the extent and the types of the deletion genes. The subjects were five Japanese X-linked CGD patients estimated to have large base deletions of 1 kb or more in the *CYBB* gene (four male patients, one female patient) and the mothers of four of those patients. The five Japanese patients were found to range from a patient exhibiting deletions only of the *CYBB* gene to a female patient exhibiting an extensive DNA deletion and the DMD and CGD phenotype manifested. Of the other three patients, two exhibited *CYBB*, *XK*, and *DYNLT3* gene deletions. The remaining patient exhibited both a deletion encompassing DNA subsequent to the *CYBB* region following intron 2 and the *DYNLT3* gene and a complex copy number variation involving the insertion of an inverted duplication of a region from the centromere side of *DYNLT3* into the deleted region.

## Introduction

Chronic granulomatous disease (CGD [OMIM 306400]) is caused by a reduced nicotinamide adenine dinucleotide phosphate (NADPH) oxidase complex deficiency and is a primary immunodeficiency that impairs the bacteria-killing ability of phagocytes in the innate immune system and occurs with an incidence of 1 in 200,000 births per year [1]. The NADPH oxidase complex is localized in the cell membrane and is composed of heterodimeric membrane-bound flavocytochrome subunits formed from *gp91^phox^* (phox for phagocyte oxidase [OMIM 300481]) and *p22^phox^* (OMIM 233690) and cytoplasmic subunits formed from *p47^phox^* (OMIM 233700), *p67^phox^* (OMIM 233710), *p40^phox^*, and *Rac2* (OMIM 602049). Reactive oxygen species (ROS) required to kill microorganisms, such as superoxide anions (O_2_^•−^) and hydrogen peroxide, are produced by the transfer of electrons from NADPH to oxygen molecules. In CGD, however, since the NADPH oxidase complex is defective or dysfunctional, it is not possible to kill microorganisms, although the leukocyte phagocytoses the microorganisms, and this leads to repeated infections by fungi and catalase-positive bacteria, such as Staphylococcus, Pseudomonas aeruginosa, Escherichia coli, and Klebsiella pneumoniae, and frequently causes fatal granulomatous inflammation.

Mutations in genes for any of the six subunits of NADPH oxidase cause CGD, but almost 2/3 of CGD cases are caused by mutations in the X-linked *CYBB* gene, which codes for *gp91^phox^*. Other CGD patients all show autosomal recessive inheritance patterns. *CYBB* is located at Xp21.1 and is a 33.4 kb gene made up of 13 coding exons and a promoter region mainly expressed in phagocytes. Its mutations have been reported to include non-sense mutations, missense mutations, splice site mutations, duplications, deletions, and insertions [2]. In Japan, more than 260 patients with CGD have been reported [3]. Genetic analysis of the 92 patients with *CYBB* gene mutations was performed. Mutations similar to those in the above reports were found, but in five of the 16 cases exhibiting deletions, the deletion was estimated to be large at 1 kb or more. Quite recently, details have been revealed from two patients of five exhibiting contiguous gene syndrome (CGS) associated with deletions of the *XK* gene responsible for McLeod syndrome (OMIM 314850) and the ornithine transcarbamylase (*OTC*) gene responsible for OTC deficiency (OTCD [OMIM 311250]) [4].

However, it is often difficult to detect multiple exon deletions and multigene deletions with conventional PCR-SSCP analysis and DNA sequencing, hindering the precise search for gene duplications and the detection of heterozygous deletions and duplications in female carriers. In addition, it is technically difficult to use Southern blot analysis for high precision, extensive deletion searches. Because of this, the details of the types of genomic rearrangements including duplications and deletions (genomic copy-number losses and gains) and their ranges (sizes and boundaries) in X-linked CGD caused by mutations in the *CYBB* gene are largely unresolved.

Array comparative genomic hybridization (aCGH) is a method that has been successfully adapted to the detection of copy number variations (CNVs) in OTCD and Duchenne/Becker muscular dystrophy (DMD [MIM 310200]), has been confirmed to show high sensitivity compared with traditional methods currently available, and has been developing rapidly in recent years [5], [6]. In the present study, we analyzed genomic rearrangements in Japanese X-linked CGD patients using aCGH analysis, revealing the extent and the types of the deletion genes.

## Results

### Search for the *CYBB* gene and *XK* gene by PCR

**Figure 1:**
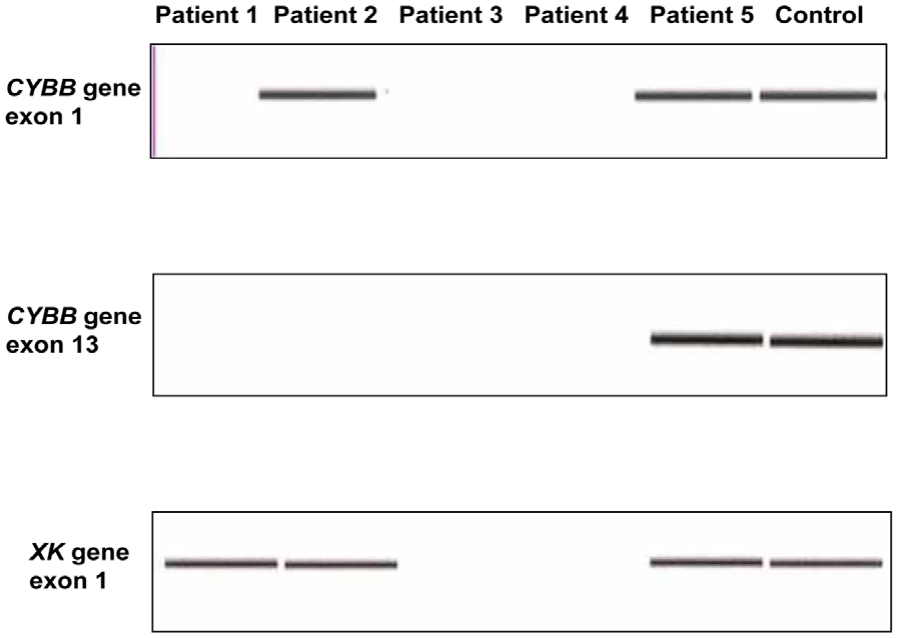
PCR Amplification Results for Exons 1 and 13 of the Chronic Granulomatous Disease Gene *CYBB* and Exon 1 of the McLeod Syndrome Gene *XK* Region. The analysis used the Agilent 2100 Bioanalyzer. Patients 1 to 5 were juvenile patients with chronic granulomatous disease, and genes from normal boys were amplified as a control. *CYBB* exon 1 was detected only from patients 2 and 5, and *CYBB* exon 13 only from patient 5. In addition, *XK* exon 1 was detected in patients 1, 2 and 5.

In patients 1, 3, and 4, neither exon 1 nor exon 13 of the *CYBB* gene was detected. In patient 2, exon 1 of the *CYBB* gene was detected, but exon 13 was not. In patient 5, both exon 1 and exon 13 of the *CYBB* gene were detected. A search for the *XK* gene was performed. Exon 1 of the *XK* gene was detected in patients 1, 2, and 5, but not in patient 3 or 4. In the healthy controls, on the other hand, exons 1 and 13 of the *CYBB* gene and exon 1 of the *XK* gene were clearly detected (Figure 1). These results suggested that while genes are present upstream of the *XK* gene in patient 1, at the very least, all *CYBB* gene exons are deleted, and that extensive deletions encompassing the *CYBB* gene and *XK* gene are exhibited in patients 3 and 4. Deletion of *CYBB* exon 13 and the centromere side was suspected in patient 2. Patient 5 exhibited a similar gene amplification pattern to the healthy controls, and no obvious abnormality was observed.

### Deletion search by aCGH

**Figure 2:**
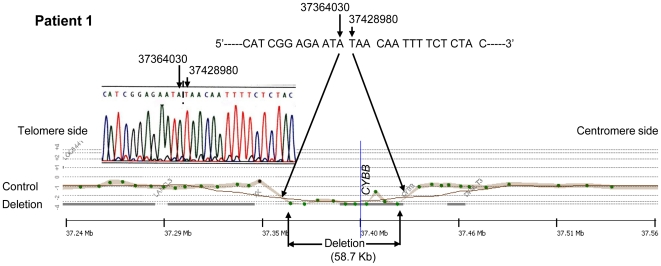
The Results of aCGH and Direct Sequence Analysis Spanning the Deletion Breakpoints in Patient 1. A female's DNA was used for the control DNA. aCGH analysis showed the deletion region to be 58.7 kb and direct sequencing after amplification by PCR showed that genes had been deleted from 37364030 to 37428980 (UCSC hg17 May.2004) and both breakpoints were bound.

**Figure 3:**
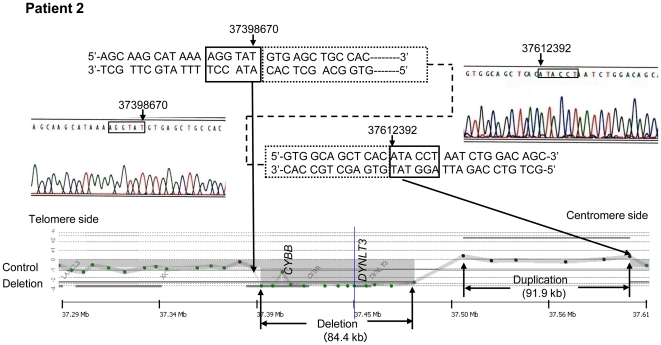
The Results of aCGH and Direct Sequence Analysis Spanning the Deletion Breakpoints in Patient 2. A female's DNA was used for the control. According to aCGH, the deletion site was 84.4 kbp and the duplication site 91.9 kb. According to the DNA walking analysis by PCR, a breakpoint was located at 37398670 of *CYBB* intron 2. Furthermore, six bases of the gene at 37612392 (UCSC hg17 May.2004) of ATACCT were bound inverted at the breakpoint, and complex structural abnormalities showing gene deletions on the inner side and duplication of the inverted part were observed.

**Figure 4:**
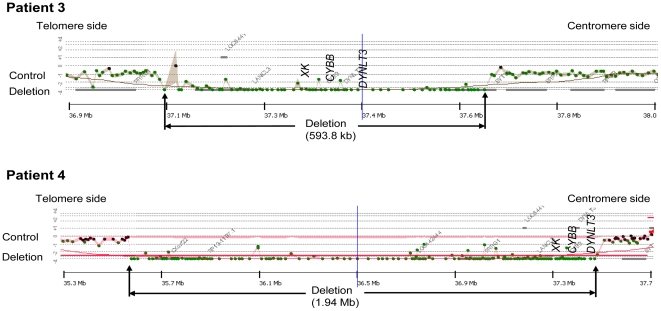
The Results of aCGH Analysis Spanning the Deletion Breakpoints in Patients 3 and 4. A female's DNA was used for the control DNA for patient 3, and a male's for patient 4. These male infants suffered deletions of three genes: the chronic granulomatous disease gene (*CYBB*), the McLeod syndrome gene (*XK*), and *DYNLT3*. The gene deletions in each patient were found to be 0.59 Mb and 1.94 Mb, respectively.

**Figure 5:**
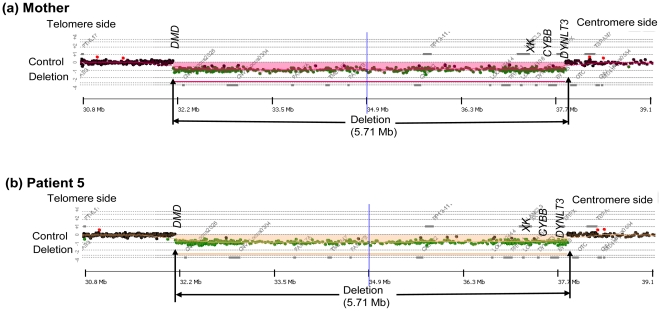
The Results of aCGH Analysis Spanning the Deletion Breakpoints in Mother and Patient 5. A female's DNA was used for the control DNA. This case involved deletions of four genes, the chronic granulomatous disease gene (*CYBB*), the Duchenne muscular dystrophy gene (*DMD*), the McLeod syndrome gene (*XK*), and *DYNLT3*. The gene deletion region was about 5.71 Mb and encompassed an area from *CYBB* to the greater part of the *DMD* (b). A similar gene deletion was also observed in the mother (a).

The results of the search for CNVs by aCGH are summarized in Table 1. Deletions were observed in all five patients. Patient 1 showed the smallest deletion of the five patients at 58.7 kb, starting upstream of the *CYBB* gene and including all the exons of the 33.4 kb *CYBB* gene (Figure 2). Patient 2 was shown to exhibit a complex CNV involving a deletion of 84.4 kb encompassing the region downstream of exon 1 of the *CYBB* gene and the *DYNLT3* gene base sequence and a base sequence duplication of 91.9 kb adjacent to the centromere side of the deletion region (Figure 3). Patients 3 and 4 showed large deletions of 0.59 Mb and 1.94 Mb, respectively, encompassing the *XK* and *CYBB* genes and the *DYNLT3* gene (OMIM 300302), the function of which is not yet clear (Figure 4). Patient 5 showed an extensive deletion (5.71 Mb), exhibiting deletion of exons 1 to 42 of the 79 exons making up the *DMD* gene in addition to deletion of the *DYNLT3* gene, *CYBB* gene, and *XK* gene (Figure 5). aCGH of the mother of patient 5 also revealed a deletion of the same extent as in patient 5. The size of the genomic DNA deletions is half of that in the healthy female controls. Specifically, this shows that patient 5 has a deletion in the maternally inherited X-chromosome. With the exception of the mother of patient 4, who could not be tested, the mothers of the other four patients all had the same CNVs as their children, and the CNVs in patient 4 were heritable, rather than de novo (Table 1).

**Table 1:** The Results of aCGH Analysis of the Length of the Gene-Deleted Region and Disease Genes.

Patient	Sex	Deletion gene	Genotype (UCSC hg17 May 2004)	aCGH Deletions size (kb)	mother
1	M	*CYBB*	chrX:g.(37367763_37367821)_(37426362_37426421) del	58.7	carrier
2	M	*CYBB, DYNLT3*	chrX:g.(37402945_37403004)_(37487331_37487390) del	84.4	carrier
			chrX:g.(37514480_37514539)_(37606312_37606370) dup	91.9	
3	M	*XK, CYBB, DYNLT3*	chrX:g.(37128233_37128292)_(37722014_37722073) del	593.8	carrier
4	M	*XK, CYBB, DYNLT3*	chrX:g.(35599031_35599090)_(37543467_37543526) del	1944.5	-
5	F	*DMD, XK, CYBB, DYNLT3*	chrX:g.(32167387_32167446)_(37878746_37878805) del	5711.4	carrier

CYBB, cytochrome b-245, beta polypeptide; XK, X-linked Kx bood group gene; DMD, Duchenne muscular dystrophy gene; DYNLT3, dynein, light chain, Tctex-type3.

### Breakpoint analysis

A common TA base sequence was observed at breakpoints in patient 1 and the deletion of chrX:g.37364030_37428980del64950 (UCSC hg17 May 2004) was observed (Figure 2). The breakpoint at the telomere side in patient 2 was shown by DNA walking analysis to be chrX:g.37398670 in intron 2 of the *CYBB* gene (Figure 3). The base sequence detected at the breakpoint was 5′-AGGTAT|GTGAGCTGCCAC---- 3′ (| denotes the breakpoint). A search using NCBI Blast Human Sequences showed that this base sequence corresponded to a sequence inversion observed from chrX:g.37612397 on the telomere side of the complementary strand. On the other hand, a search in the neighborhood of chrX:g.37612392, thought to be the breakpoint on the centromere side, found the normal 5′-GTGGCAGCTCAC|ATACCTAATCTGGACAGC----3′ chromosomal base sequence. Because of this, it is thought that a 91.9 kb base sequence duplication as well as the deletion of an 84.4 kb base sequence shown by the results including aCGH analysis occurred in patient 3, and that since the duplicate centromere side end had the sequence 5′-AGGTAT-3′ on the complementary strand, one of the duplicate strands must have inverted and recombined with the common 5′-AGGTAT-3′ sequence at the telomere side of the 5′ breakpoint of the 84.4 kb deletion. However, although the base sequence of the binding region thought to be present between the duplicate strands was investigated by the step-by-step PCR method and DNA walking analysis, it is as yet unclear.

### Investigation of skewed lyonization by methylation specific PCR

**Figure 6:**
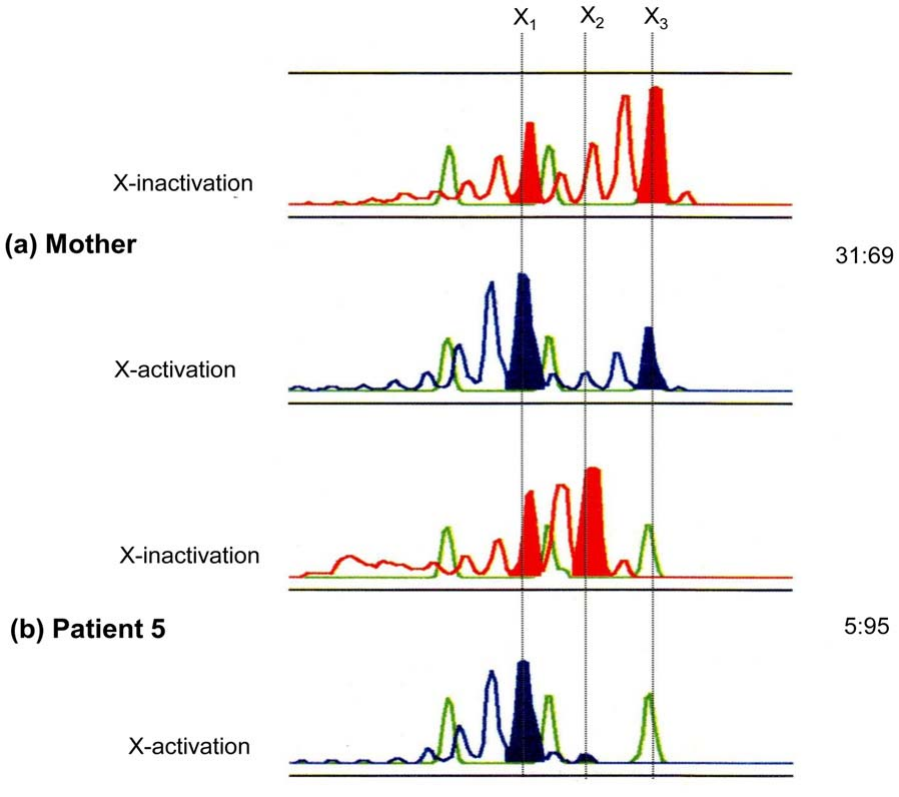
The Results of an Analysis of X-Chromosome Inactivation and Activation Patterns in the Chronic Granulomatous Disease Sufferer Patient 5 and Her Mother. The female infant had inherited an abnormal (with deletion) X-chromosome X1 from the mother and a normal X-chromosome X2 from the father. A random inactivation pattern of 69∶31 was observed in the mother (carrier). In the female patient, however, a markedly skewed X-chromosome inactivation pattern of 95∶5 (with activation of the X-chromosome with gene deletions transmitted from the mother and inactivation of the X-chromosome with the normal genes transmitted from the father) was observed.

The analysis was conducted using peripheral blood mononuclear cells (PBMCs) from patient 5 and her mother. An abnormal (with deletion) X-chromosome from the mother (X1: 186 bp allele) and a normal X-chromosome from the father (X2: 195 bp allele) had been inherited. In the patient, a markedly skewed inactivation pattern was observed in which X-chromosomes from which genes had been deleted were activated and X-chromosomes with normal gene alleles were inactivated (Figure 6).

## Discussion

The aCGH analysis showed gene deletions of various sizes ranging from 58.7 kb to 5.71 Mb. In patient 1, only the *CYBB* gene was deleted, whereas CGS associated with simultaneous deletions of adjacent genes occurred in the other patients.

In patients 1 and 2, precise breakpoints were analyzed by direct sequencing methods after amplification with PCR and DNA walking. Analysis of four patients including two patients^4^ previously reported by us found two cases in which all genes within the breakpoints were deleted and one case each of complex structural abnormalities associated with gene insertions and further inversions in addition to deletions. The ends in four patients had no gene sequences expected to result in a common breakpoint.

Chromosomal deletions require a search over the entire range since variation in extent may produce false negatives by PCR and duplication of other regions may be involved. Conventionally, chromosome banding and fluorescence in situ hybridization (FISH) [7]–[9] have been used for analysis of chromosomal deletions, duplications, and translocations, but with chromosome banding, the detection limit is 1 Mb or more even in analyses with well-resolved prometaphase chromosomes. On the other hand, a combination of PCR and automated fluorescent DNA sequencing allows analysis of single base mutations on the chromosome and offers the highest resolution. However, the length of the deletion varies depending on the patient and for larger deletions, it is difficult to set primers. In addition, when no PCR products are generated, it is thought that nucleotide insertion into the deletion site or mutations in the base sequence at the primer binding site may have occurred. In recent years, aCGH has been developed and come into use as a method of analyzing CNVs on chromosomes. In principle, aCGH is a method of adhering a human gene-specific oligonucleotide probe to a glass slide and competitively hybridizing it with patient and control DNA, each labeled with different fluorochromes, and detecting deletions in the genome and amplification at a resolution of several kb by measuring the fluorescence. But while this method has high specificity and high resolution, it has the disadvantage including difficulty in identifying chromosomal translocations.

Deletions due to genomic mutations are often caused by tandem repeats and interspersed repeats. Interspersed repeats in particular include shorts direct repeats, interspersed repeat elements (e.g. Alu repeats), inverted repeats, low copy number long repeats, and active transposable elements and are prone to large scale deletions and duplications [10], [11]. In patient 1, the entire *CYBB* gene within the breakpoints was deleted. The *CYBB* gene in patient 2 was found up to exon 2 and entirely deleted from exon 3. Moreover, it displayed a complex structure in which the breakpoint chrX:g.37398670 bound to the base sequence inverted from 37612392 and the region downstream of 37612392 (UCSC hg17 May. 2004) exhibited a successive existing base sequence. Alu sequences, which existed the most, were observed in the neighborhood of the ends, but were not directly involved in the breakpoints.

Patient 5 was female but was diagnosed with CGD, having a history of increased susceptibility to infection and confirmed as 46, X del (X) (p 21.1 p.21.2) on chromosome banding. aCGH showed that the mother was a carrier with an X-chromosome with the same deletion as the patient. The existence of skewed lyonization was investigated by analysis of X-chromosome inactivation on the basis of DNA methylation [12]. In the analysis of the patient and her mother, she had inherited an abnormal X-chromosome (X1: 186 bp allele) from her mother and a normal X-chromosome (X2: 195 bp allele) from her father. A markedly skewed inactivation pattern was observed in the patient, where the X-chromosome with deleted genes was activated and the X-chromosome having the normal genetic allele was inactivated (Fig. 6). In consideration of these results and of the measured percentage of gp91-positive cells in the patient's neutrophils (markedly low at about 5%) in addition to the notably decreased ability to produce ROS when measured by the NBT assay (as described later in the “Materials and Methods” section), the normal X-chromosome was thought to be inactivated in the majority of cells of this patient, which was believed to be a factor at the onset of the disease (a symptomatic carrier). Each cell expresses alleles from only one X-chromosome. Reports on skewed lyonization include Bruton tyrosine kinase (BTK) deficiency (OMIM 300300) [13], DMD [14] and CGD [15]–[17], however, CGS associated with deletions to the same wide extent as in this patient has not been observed. Francke et al. [18] reported the case of a male infant in which CGD with extensive X-chromosome deletions was complicated by DMD, retinitis pigmentosa, and mental retardation. Here, we analyzed the case of a female infant who exhibited a similar phenotype with an extensive genomic deletion and revealed that the X-linked recessive disorder of women was caused by skewed lyonization.

While chromosomal deletions, inversions, and duplications occur singly or in combination, reciprocal recombinations due to translocations have been reported [19]–[21]. In patient 1, it was speculated that gene rearrangements due to non-homologous end- joining (NHEJ) showing the repeated sequence of only two bases (TA) at the ends of the deleted gene may have occurred (Figure 7.1a, 1b). Patient 2 had a complex structural abnormality, and it was hypothesized that this may have been caused by fork stalling and template switching (FoSTeS)/microhomology-mediated break-induced replication (MMBIR) [22], [23]. In patient 2, a three base repeat sequence of AAT prone to cause changes in copy number or sequence swaps was observed in the original DNA strand that acts as a template for the lagging strand during DNA replication. This AAT is thought to bind to a complementary DNA sequence of TTA nearby on the same strand of DNA, forming a loop and inhibiting expansion of the replication fork (Figure 7.2a). Furthermore, releasing the leading strand with the free 3′ end, AGGTAT, and binding to the complementary strand ATACCT of another replication fork, a leading strand 5′-AGGTATGTGAGC----3′ was synthesized (Figure 7.2b). The gene synthesized on the leading strand is hypothesized to cause structural abnormalities leading to duplications and inversions of the DNA strand subsequent to the deleted DNA strand by a return to the lagging strand after the end of replication and the binding of the 5′---- GCTCACATACCT-3′ telomere side terminus with the normal gene to the 5′-AGGTATGTGAGC----3′ centromere side terminus gene by DNA ligase (Figure 7.2c). As far as we could find, while a breakage-fusion-bridge cycle has been reported as a mechanism for large structural abnormalities causing deletions, inversions, and duplications during chromosome division [23], no cases of structural abnormalities occurring in the vicinity of the same gene have been observed.

**Figure 7:**
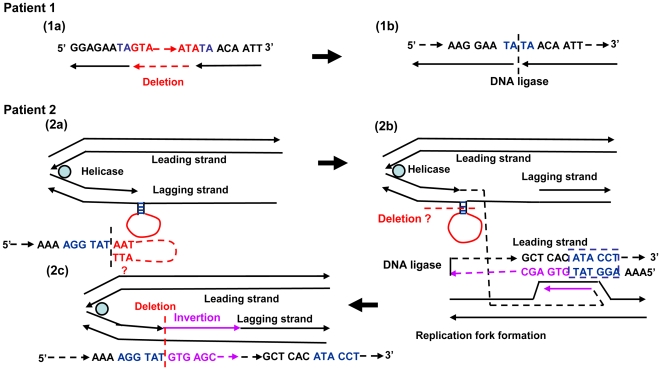
Genomic Rearrangement Mechanisms. For the gene deletions in patient 1, gene rearrangements exhibiting deletions due to non-homologous end-joining (NHEJ) were thought to have occurred, since repeated two base sequences of TA were observed at both ends of the gene (1a, 1b). In patient 2, it was hypothesized that rearrangement might have occurred through a mechanism involving a combination of fork stalling and template switching (FoSTeS)/microhomology-mediated break-induced replication (MMBIR). A three base repeat sequence of AAT prone to cause changes in copy number or sequence swaps was observed in a discontinuous end of the lagging strand during DNA replication. This formed a loop by binding to TTA on its complementary strand with replication slippage occurring (2a). At the discontinuous end, the lagging strand with the loop formed had a six base microhomology of AGGTAT and dissociated. The dissociated end subsequently bound to the ATACCT found at the duplication front of the leading strand. Synthesis and extension of the leading strand then occurred originating from the binding site (2b). The gene synthesized on the leading strand is hypothesized to cause structural abnormalities leading to duplications of the DNA strand subsequent to the DNA strand deletion by returning to the lagging strand after the end of duplication and the binding of the end that has finished duplication to the starting end of the normal gene on the lagging strand by DNA ligase (2c).

## Materials and Methods

### Subjects

The subjects were five Japanese X-linked CGD patients estimated to have large base deletions of 1 kb or more in the *CYBB* gene (four male patients, one female patient) and the mothers of four of these patients. The four male patients were diagnosed as CGD on the basis of the past history with increased susceptibility to infection, and of deficiencies in ROS productivity by the nitroblue tetrazolium test (NBT) and the neutrophil chemiluminescence method. Two of the male patients exhibited reduced expression of erythrocyte Kell blood group antigen, acanthocytosis, and also high serum CPK activity. In the female infant patient, in addition to a history of increased susceptibility to infection at the age of 11 and a marked reduction in ROS productivity to around 5% of normal, chromosome banding showed 46, X, del (X)(p21.1p21.2), and CGD was clinically diagnosed.

### DNA isolation

All the patient samples in the present study were used with the approval of the Saitama Children's Medical Center Ethics Committee and after obtaining written informed consent, and the procedures were conducted to the principles expressed in the Declaration of Helsinki, 2008. Patient's parents provided written informed consent. DNA was extracted with a DNA Blood Mini Kit (Qiagen, Valencia, CA) after isolation of heparinized PBMCs.

### PCR-band studies

*CYBB* gene and the adjacent X-linked Kx blood group-related *XK* gene were amplified by PCR and the detection of individual genes was attempted using the Agilent 2100 Bioanalyzer (Agilent Technologies, Santa Clara, CA) according to the manufacturer's instructions. The following primers were used for PCR: *CYBB* ex1: forward, 5′-AATGTGTTTTACCCAGCACG-3′ and reverse, 5′-TGCTTTGGTCTATTTTAGTTCC-3′; *CYBB* ex13: forward, 5′-TAGACATCTCATCCCAAAGC-3′ and reverse, 5′-TTATTTGAGCATTTGGCAGC-3′; *XK* ex1: forward, 5′-TTTCCCAAGATAGGACCC-3′ and reverse, 5′-GTTGAACCACAAGAAACTGC-3′.

### aCGH analysis

aCGH measurements used the Agilent Genomic DNA Labeling Kit (Agilent Technologies). In brief, 1 µg of each of the patient and control DNA (patients 1, 2, and 3: gender non-matched reference controls, patients 4 and 5: gender matched reference controls) were digested with two restriction enzymes (AluI and RsaI; Life Technologies, Carlsbad, CA). Random-primed DNA labeling was performed according to the manufacturer's recommended protocol using Cy3-dUTP for patient DNA and Cy5-dUTP for control DNA. After reacting at 37°C for 2 hrs, labeled patient and control DNA were purified using DNA Microcon YM-3-filter units (Millipore, Billerica, MA), mixed, and human Cot-1 DNA (Life Technologies) and hybridization buffer (Agilent Technologies) were added. The mixed samples were applied to microarray slides (Human Genome CGH Microarray 244A, Agilent Technologies) and the microarray slides were hybridized in Agilent SureHyb chambers at 65°C for 40 hrs. After a second washing operation and microarray scanning (Agilent DNA microarray scanner and Agilent Scan Control Software), images were extracted using the Feature Extraction Software (version 9.5.3.1; Agilent Technologies) and imported into the Agilent CGH-Analytics V3.5.14 Software for analysis, and statistically significant CNVs were determined using the aberration detection module (ADM)-2 algorithm. For details of the statistical algorithm, the Agilent Technologies user's manual is available (http://www.agilent.com/chem/goCGH). Copy-number size was measured as the difference between the first and last probe position in the region deleted using the algorithm. The chromosome resolution of DNA variants with this method averaged 6.4 kb.

### Determining of breakpoints

Breakpoints were analyzed in patients 1 and 2. Deletions of telomeric and centromeric breakpoints revealed by the aCGH results of patient 1 were determined by the previously reported step-by-step PCR method [4] using the TAKARA Ex Taq (Takara Bio, Tokyo, Japan). Finally, after amplification by PCR with primers set to sandwich the telomeric and centromeric breakpoints, direct sequence analysis (ABI 310, Life Technologies) of the PCR product was performed. In patient 2, on the other hand, as shown in the results, it was not possible to find the breakpoints by the method described above. To determine the deletion breakpoints, a DNA walking analysis was conducted according to the manufacturer's instructions (DNA Walking Kit; Seegene, Rockville, MD). For patient 2, gene-specific primary 1stF 5′-GGAATCTACTGTGGAAATGC-3′, 2ndF 5′-TGTTACATCATGCTGAAACTATG-3′, 3rdF 5′-TCCCGCCAAAATATGCAAC-3′ and nested primers designed based on the base sequence near the telomeric breakpoint region were used for PCR amplification in combination with primary and nested PCR to Genome Walker Adaptors. To confirm the results, gene-specific primes were designed to straddle the deletion breakpoints, and PCR products were amplified from genomic DNA. PCR products were analyzed by direct sequence analysis. Gene sequences obtained from the analysis were searched for genetic information using the NCBI's BLAST Human Sequences (http://www.ncbi.nlm.nih.gov/genome/seq/BlastGen/BlastGen.). Androgen Receptor in the X-chromosome (the HUMARA region) of patient 5 was analyzed by methylation specific PCR and examined for skewed lyonization [12].
